# B_12_H*_n _*and B_12_F*_n_*: planar vs icosahedral structures

**DOI:** 10.1186/1556-276X-7-236

**Published:** 2012-04-30

**Authors:** Nevill Gonzalez Szwacki, C J Tymczak

**Affiliations:** 1 Institute of Theoretical Physics, Faculty of Physics, University of Warsaw, ul. Hoża 69, Warsaw, 00-681, Poland; 2 Department of Physics, Texas Southern University, Houston, TX, 77004, USA

## Abstract

Using density functional theory and quantum Monte Carlo calculations, we show that B_12_H*_n _*and B_12_F*_n _*(*n *= 0 to 4) quasi-planar structures are energetically more favorable than the corresponding icosahedral clusters. Moreover, we show that the fully planar B_12_F_6 _cluster is more stable than the three-dimensional counterpart. These results open up the possibility of designing larger boron-based nanostructures starting from quasi-planar or fully planar building blocks.

## Background

The icosahedral B_12_H_12_^2- ^cluster is the most stable molecule among the number of polyhedral boranes synthesized so far [1]. A large-scale and efficient synthesis of fully fluorinated boron hydrides, e.g., icosahedral B_12_F_12_^2-^, has been also reported [2]. On the other hand, the all-boron *C*_3v_-B_12 _cluster is quasi-planar, and it was reported to be one of the most stable all-boron clusters. It was also established by extensive computations that the quasi-planar B_12 _cluster is much lower in energy than the all-boron icosahedral B_12 _cluster. This was reported not only for the neutral clusters [3], but also for the charged ones [4]. It is then interesting to investigate what happens with the relative stability of the two (quasi-planar and three-dimensional (3D)) all-boron structures upon addition of hydrogen or fluorine atoms. This is the purpose of this study.

Quasi-planar and 3D boron clusters with the number of hydrogen atoms smaller than the number of boron atoms have been studied both theoretically [5-11] and experimentally [12-14]. Ohishi et al. [12] reported the formation of B_12_H*_n_*^+ ^(*n *= 0 to 12) cationic clusters through ion-molecule reactions of the decaborane ions (B_10_H*_n_*^+^, *n *= 6 to 14) with diborane molecules (B_2_H_6_) in an external quadrupole static attraction ion trap. The mass spectrum analysis revealed that among the B_12_H*_n_*^+ ^clusters with different hydrogen content *n*, the B_12_H_8_^+ ^molecule was the main product. In the same study, using first principle calculations with the Becke 3-parameter Lee-Yang-Parr (B3LYP) hybrid functional and the 6-31G(d) basis set, the authors compared the relative energies of quasi-planar and 3D B_12_H*_n_*^+ ^clusters with *n *varying from 0 to 12. According to that study, two-dimensional (2D) clusters with *n *= 0 to 5 are energetically preferred over the 3D structures, whereas 3D clusters are energetically favored for *n *≥ 6. In a more recent combined experimental/theoretical study, Ohishi et al. [14] suggested that quasi-planar B_12_H*_n_*^+ ^with *n *= 0 to 3 clusters can be obtained by further removal of H atoms from the decaborane ions. This opens up the possibility of changing the structure of the B_12_H*_n_*^+ ^cluster by controlling the number of hydrogen atoms in the cluster.

To our knowledge, there are no previous studies on the structure and properties of quasi-planar B_12_F*_m _*clusters. However, the structures of two polyboron fluorides, B_8_F_12 _and B_10_F_12_, revealing unusual open structures were recently determined [15].

## Methods

The initial search for the most stable structures of the boron hydrides B_12_H*_n _*and boron fluorides B_12_F*_n _*was done at the B3LYP/6-31G(d) level of theory using the FreeON code [16] with no symmetry restrictions. For clusters with an even number of hydrogen or fluorine atoms (even number of electrons), the computations were performed for the singlet multiplicity only, whereas doublet and quartet multiplicities were considered for clusters with an odd number of hydrogen or fluorine atoms (odd number of electrons). In the later case, structures with lower multiplicity were energetically more favorable. For the charged structures, a similar analysis has been done, and the ground state was found to have the lowest multiplicity. Next, the low-lying isomers of B_12_H*_n _*and B_12_F*_n _*have been re-optimized using the GAMESS-US code [17] at the B3LYP/6-311++G(d, p) level of theory, and for the resulting structures, the vibrational analysis has been done to identify true local minima. It is important to mention that the 'bare' B_12 _icosahedron undergoes distortions after structural optimization and that its symmetry is *S*_2 _[3], not *I*_h_. However, we will refer to that structure and its derivatives as icosahedral or 3D. The quasi-planar or fully planar clusters, for a change, will be often labeled as 2D structures.

The nucleus independent chemical shift (NICS) values and magnetic susceptibility tensors were calculated using the Gaussian 03 package [18] at the B3LYP/6-311++G(d, p) level of theory. To obtain the NICS values, we have used the gauge-independent atomic orbital method, and the magnetic susceptibility tensors were calculated using the continuous set of gauge transformations method.

The quantum Monte Carlo calculations have been done using the QWalk [19] package in two steps. The first step involved optimizing the trial many-body wave function by doing variational Monte Carlo calculations. The trial wave function was of the Slater-Jastrow form. The Slater determinants were constructed using B3LYP orbitals, generated using the GAMESS-US code with the previously optimized geometries within the B3LYP/6-311++G(d, p) level of theory. For the calculations, we have used Gaussian basis sets with effective core potentials [20]. In the second step, we have done fixed-node diffusion Monte Carlo (DMC) calculations with the previously optimized trial wave functions. In the computations, we have used a time step of 0.005 a.u. The DMC error bars are about 0.1 eV.

## Results and discussion

### Planar vs icosahedral structures

**Figure 1:**
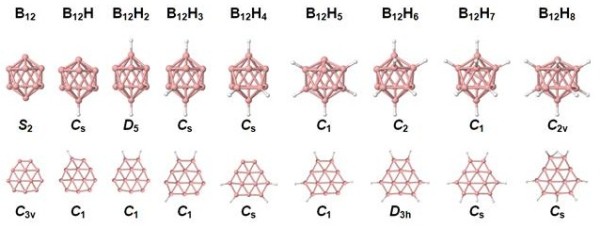
**The most stable structures of 3D and 2D B_12_H*_n _*(*n *= 0 to 8) clusters**. The symmetry of each cluster is given.

**Figure 2:**
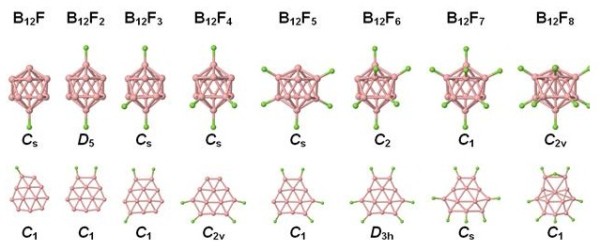
**The most stable structures of 3D and 2D B_12_F*_n _*(*n *= 1 to 8) clusters**. The symmetry of each cluster is given.

The procedure for determining the most stable isomers of B_12_H*_n _*was very similar to that reported in [12], namely, we started with optimized icosahedral and quasi-planar B_12 _clusters, and for a given *n*, we have calculated the total energies of all possible clusters that resulted from adding hydrogen atoms to the vertices of the distorted icosahedron or to the outer boron atoms of the quasi-planar structure; 2D clusters with an even *n *have been considered in our previous work [6], and here, we have extended the investigation to an odd *n*. The energetically most favorable 2D and 3D B_12_H*_n _*structures are shown in Figure 1. The minimum-energy cluster structures of B_12_F*_n_*, shown in Figure 2, have been found by replacing the hydrogen atoms of the low-lying B_12_H*_n _*isomers by fluorine atoms. Interestingly enough, the resulting structures are similar to those found for B_12_H*_n_*. One of the small differences is that the B-F bonds are on average 13% longer than the B-H bonds.

**Figure 3:**
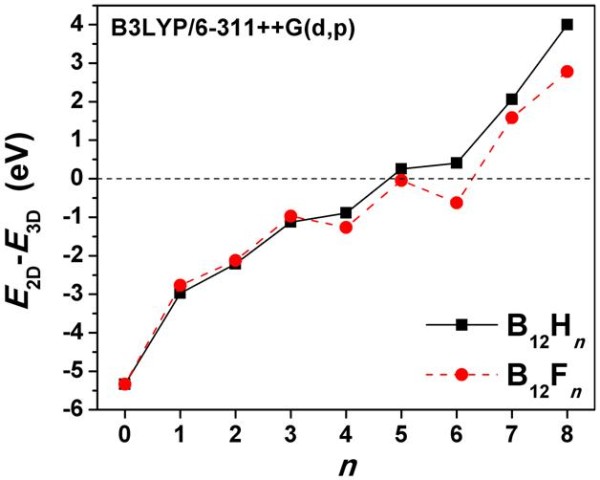
**Energy difference between quasi-planar and icosahedral B_12_X*_n _*(X = H, F) clusters as a function of number atoms**.

In Figure 3, we have plotted the total energy difference between quasi-planar (or fully planar) and icosahedral B_12_X*_n _*(X = H, F) clusters as a function of *n*, the number of H or F atoms in the cluster. As can be seen from the figure, the quasi-planar clusters with up to four hydrogen atoms are more stable than the corresponding icosahedral structures (a similar result has been recently reported [11] for B_12_H*_n_*^0/- ^clusters). The same is true for the fully planar B_12_F_6 _molecule, which is 0.63 eV lower in energy than the 3D cluster. The 2D and 3D B_12_F_5 _isomers are almost degenerated in energy. From Figure 3, we can also see that the energy difference, *E*_2D _- *E*_3D_, increases monotonically with *n*, with the exception of the two 'minima' for B_12_F_4 _and B_12_F_6_. These two minima may suggest an additional stabilization of the 2D structures over the 3D counterparts due to the presence of aromatic stabilization energy.

Similar results to those presented in Figure 3 were reported for the icosahedral and quasi-planar B_12_H*_n_*^+ ^structures [12]. However, in their recent work, Ohishi et al. [14] have used the PBE0 functional instead of the B3LYP functional to determine the energies of the B_12_H*_n_*^+ ^clusters. The authors' choice was motivated by the fact that the B3LYP functional may overestimate the energy difference between 2D and 3D structures. To address this problem, we calculated the energy difference between the 2D and 3D structures of B_12_, B_12_H_6_, and B_12_F_6 _using the very accurate DMC approach. The DMC *E*_2D _- *E*_3D _values are -5.13, 0.79, and -0.47 eV for B_12_, B_12_H_6_, and B_12_F_6_, respectively, whereas the corresponding B3LYP values are -5.34, 0.41, and -0.63 eV, respectively (see Figure 3). This means that the DMC values are shifted up by a value not larger than about 0.4 eV with respect to the B3LYP values. This, however, does not affect the conclusions that are drawn from Figure 3, since even if we shift up the curves by 0.4 eV, the quasi-planar B_12_H*_n _*and B_12_F*_n _*(*n *= 0 to 4), and the fully planar B_12_F_6 _clusters still remain energetically favorable.

### Fully planar clusters: B_12_H_6 _vs B_12_F_6_

As calculated here and also reported in [11], the fully planar B_12_H_6 _cluster corresponds to a local minimum of energy, whereas the *D*_3h_-B_12_F_6 _structure wins the competition with other 2D and 3D isomers and corresponds to a global minimum of energy. Many properties of the B_12_H_6 _cluster have been previously described in [6], but for consistency purposes, we have repeated some of those calculations at the B3LYP/6-311++G(d, p) level of theory. The highest occupied molecular orbital-lowest unoccupied molecular orbital (HOMO-LUMO) gaps of the planar B_12_H_6 _and B_12_F_6 _structures are 3.54 and 4.39 eV, respectively, whereas the HOMO-LUMO gaps of the corresponding 3D clusters are the same and equal to 2.73 eV. The B-H and B-F interatomic distances are 1.179 and 1.326 Å in B_12_H_6 _and B_12_F_6_, respectively. For comparison, the computed B-H and B-F bond lengths in borane (BH_3_) and boron trifluoride (BF_3_) are 1.190 and 1.318 Å, respectively.

While both 2D structures, B_12_H_6 _and B_12_F_6_, have similar shape and size, they exhibit quite different magnetic properties that are directly related to aromaticity. First, we have computed the anisotropy of magnetic susceptibility. The values for B_12_H_6 _and B_12_F_6 _are -208.1 and -125.8 cgs ppm, respectively. The isotropic values of the magnetic susceptibility are -91.9 and -118.2 cgs ppm for B_12_H_6 _and B_12_F_6_, respectively. These results suggest that the induced ring current is stronger for B_12_H_6 _than for B_12_F_6_. Similarly, as reported in [6] for B_12_H_6_, the central part of the B_12_F_6 _molecule has a paratropic current flowing inside the inner B_3 _triangle. The antiaromaticity of the inner triangle is, however, smaller for B_12_F_6 _than for B_12_H_6 _since the NICS(0) values are 3.9 and 13.3 ppm, respectively. A global aromatic current is dominant above the B_12_F_6 _(B_12_H_6_) molecule since the NICS values are negative, NICS(1) = -5.5 ppm (-3.6 ppm) and NICS(2) = -4.8 ppm (-5.0 ppm), 1 and 2 Å above and below the center of the cluster.

**Figure 4:**
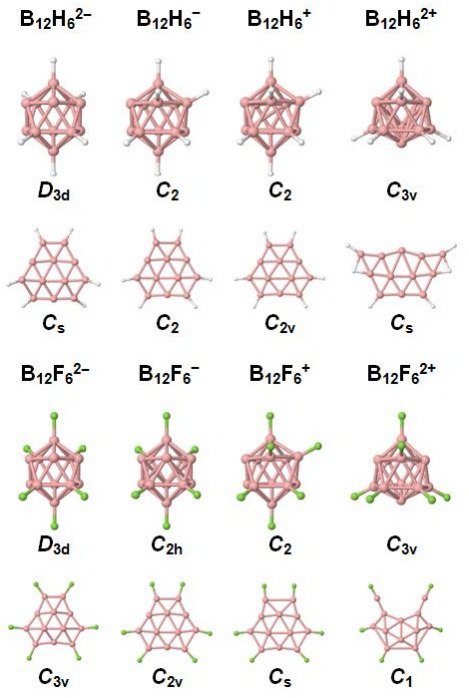
**The structures of charged 3D and 2D clusters of B_12_H_6 _and B_12_F_6_**. The symmetry of each cluster is provided.

**Figure 5:**
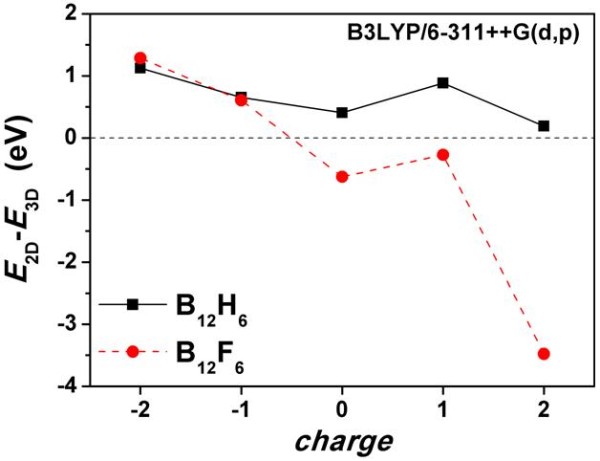
**Energy difference between quasi-planar and icosahedral [B_12_X_6_]*^q ^*(X = H, F) clusters as a function of cluster charge**.

To examine the influence of charge on the structure and stability of the fully planar clusters, we have also studied charged 2D and 3D B_12_X_6 _(X = H, F) structures. The lowest energy 2D structures identified for B_12_H_6 _and B_12_F_6 _were used as initial structures for the structural optimization at a given charge state. For the 3D structures, we have made a search over all possible configurations of the hydrogen or fluorine atoms. For the lowest energy structures with an even number of electrons, a singlet multiplicity has been assumed, whereas doublet and quartet multiplicities were considered for clusters with an odd number of electrons. In the later case, clusters with lower multiplicity were energetically more favorable. The structures of the 2D and 3D charged clusters are shown in Figure 4. It has been previously reported that the fully planar *D*_3h_-B_12_H_6 _cluster undergoes structural distortions if charged with one electron, although the quasi-planarity is preserved [6]. In general, all the charged 2D B_12_X_6 _(X = H, F) clusters are quasi-planar rather than fully planar, as can be seen in Figure 4. In Figure 5, we have plotted the energy difference between 2D and 3D [B_12_X_6_]*^q ^*(X = H, F) structures as a function of the cluster charge state *q*. We have found that the addition of one or two electrons to fully planar B_12_H_6 _and B_12_F_6 _clusters (or the removal of one electron from them) makes those structures even less energetically favorable with respect to the corresponding 3D isomers. This is, however, less pronounced for B_12_H_6 _than for B_12_F_6 _as shown in Figure 5. Finally, it should be noted that the quasi-planar B_12_F_6_^2+ ^cluster is much more stable than its 3D isomer. Finally, all structures and energies are provided in Additional file 1.

## Conclusions

Our density functional theory and quantum Monte Carlo results show that the B_12_H*_n _*and B_12_F*_n _*(*n *= 0 to 4) quasi-planar structures are energetically more favorable than the corresponding icosahedral clusters and that the fully planar B_12_F_6 _cluster is more stable than the 3D counterpart. We have also shown that negative or positive charge further stabilizes the 3D over the 2D B_12_X_6 _(X = H, F) clusters (except for B_12_X_6_^2+^, where the opposite is observed). Our findings are potentially useful for designing larger boron-based nanostructures starting from quasi-planar or fully planar building blocks.

## Competing interests

The authors declare that they have no competing interests.

## Authors' contributions

NGS conceived the study, did the calculations, and drafted the manuscript. CJT conceived the study and revised the manuscript. All authors read and approved the final manuscript.

## Supplementary Material

Additional file 1 **Total electronic energies of the boron structures**. Electronic supplementary material Figure S1 shows a fully planar boron-based nanostructure, B_504_H_36_, which was obtained starting from planar B_12_H_6 _building blocks. Table S1 recollects total energies and Cartesian coordinates of the optimized structures shown in Figures 1 and 2. Click here for file

## References

[B1] Pitochelli AR Hawthorne FM The isolation of the icosahedral B_12_H_12_^-2 ^Ion J Am Chem Soc 1960 82 3228 3229

[B2] Peryshkov DV Popov AA Strauss SH Direct perfluorination of K_2_B_12_H_12 _in acetonitrile occurs at the gas bubble - solution interface and is inhibited by HF. Experimental and DFT study of inhibition by protic acids and soft, polarizable anions J Am Chem Soc 2009 131 18393 18403 10.1021/ja9069437 19954188

[B3] Atiş M Özdoğan C Güvenç ZB Structure and energetic of B_n _(n = 2-12) clusters: electronic structure calculations Int J Quantum Chem 2007 107 729 744 10.1002/qua.21171

[B4] Zhai HJ Kiran B Li J Wang LS Hydrocarbon analogues of boron clusters - planarity aromaticity and antiaromaticity Nat Mater 2003 2 827 833 10.1038/nmat1012 14608377

[B5] Alexandrova A Koyle E Boldyrev A Theoretical study of hydrogenation of the doubly aromatic B_7_^- ^cluster J Mol Model 2006 12 569 576 10.1007/s00894-005-0035-5 16261298

[B6] Gonzalez Szwacki N Weber V Tymczak CJ Aromatic borozene Nanoscale Res Lett 2009 4 1085 1089 10.1007/s11671-009-9362-2 20596438 PMC2893872

[B7] Sahu S Shukla A Probing aromaticity of borozene through optical and dielectric response: a theoretical study Nanoscale Res Lett 2010 5 714 719 10.1007/s11671-010-9536-y 20672056 PMC2894153

[B8] Forte G La Magna A Deretzis I Pucci R Ab initio prediction of boron compounds arising from borozene: structural and electronic properties Nanoscale Res Lett 2010 5 158 163 10.1007/s11671-009-9458-8 20652134 PMC2893925

[B9] Chen Q Li S-D π-Aromatic B_16_H_6_: a neutral boron hydride analogue of naphthalene J Clust Sci 2011 22 513 523 10.1007/s10876-011-0400-8

[B10] Böyükata M Güvenç ZB DFT study of Al doped cage B_12_H_n _clusters Int J Hydrogen Energ 2011 36 8392 8402 10.1016/j.ijhydene.2011.04.078

[B11] Bai H Li S-D Hydrogenation of B_12_^0/-^: a planar-to-icosahedral structural transition in B_12_H_n_^0/- ^(n = 1-6) boron hydride clusters J Clust Sci 2011 22 525 535 10.1007/s10876-011-0408-0

[B12] Ohishi Y Kimura K Yamaguchi M Uchida N Kanayama T Formation of hydrogenated boron clusters in an external quadrupole static attraction ion trap J Chem Phys 2008 128 124304 124307 10.1063/1.2894864 18376916

[B13] Ohishi Y Kimura K Yamaguchi M Uchida N Kanayama T Energy barrier of structure transition from icosahedral B_12_H_6_^+ ^to planar B_12_H_5_^+ ^and B_12_H_4_^+ ^clusters J Phys Conf Ser 2009 176 012030

[B14] Ohishi Y Kimura K Yamaguchi M Uchida N Kanayama T Synthesis and formation mechanism of hydrogenated boron clusters B_12_H_n _with controlled hydrogen content J Chem Phys 2010 133 074305 074307 10.1063/1.3474996 20726640

[B15] Pardoe JAJ Norman NC Timms PL Parsons S Mackie I Pulham CR Rankin DWH The surprising structures of B_8_F_12 _and B_10_F_12_ Angew Chem Int Edit 2003 42 571 573 10.1002/anie.200390164 12569493

[B16] Bock N Challacombe M Gan CK Henkelman G Nemeth K Niklasson AMN Odell A Schwegler E Tymczak CJ Weber V FreeON http://freeon.org/

[B17] Schmidt MW Baldridge KK Boatz JA Elbert ST Gordon MS Jensen JH Koseki S Matsunaga N Nguyen KA Su S Windus TL Dupuis M Montgomery JA GAMESS-US, version 1 OCT 2010 (R1) http://www.msg.chem.iastate.edu/gamess/

[B18] Frisch MJ Trucks GW Schlegel HB Scuseria GE Robb MA Cheeseman JR Montgomery JA Jr Vreven T Kudin KN Burant JC Gaussian 03, revision E.01 http://www.gaussian.com/

[B19] Wagner LK Bajdich M Mitas L QWalk, version 0.95.0 http://www.qwalk.org/

[B20] CRENBL ECP http://bse.pnl.gov/

