# Supplementary material for: B12Hn and B12Fn: planar vs icosahedral structures
Source: Nanoscale Res Lett. 2012 Apr 30;7(1):236. doi: 10.1186/1556-276X-7-236 (PMC3359178; doi:10.1186/1556-276X-7-236)
Supplement: Additional file 1 — Total electronic energies of the boron structures. Electronic supplementary material Figure S1 shows a fully planar boron-based nanostructure, B504H36, which was obtained starting from planar B12H6 building blocks. Table S1 recollects total energies and Cartesian coordinates of the optimized structures shown in Figures 1 and 2. [file 1556-276X-7-236-S1.DOC]

Electronic Supplementary Material

**B_12_H*_n_* and B_12_F*_n_*: Planar *vs* Icosahedral Structures**

N. Gonzalez Szwacki^1^ and C. J. Tymczak^2*^

*^1^Institute of Theoretical Physics, Faculty of Physics, University of Warsaw, ul. Hoża 69, 00-681 Warsaw, Poland*

^2^Department of Physics, Texas Southern University, Houston, Texas 77004, USA

^*^ Address correspondence to tymczakcj@tsu.edu

***Figure S1***. The structure of B_504_H_36_ optimized at the B3LYP/STO-3G level of theory. This large boron based nanostructure is fully planar and was obtained starting from planar B_12_H_6_ building blocks.


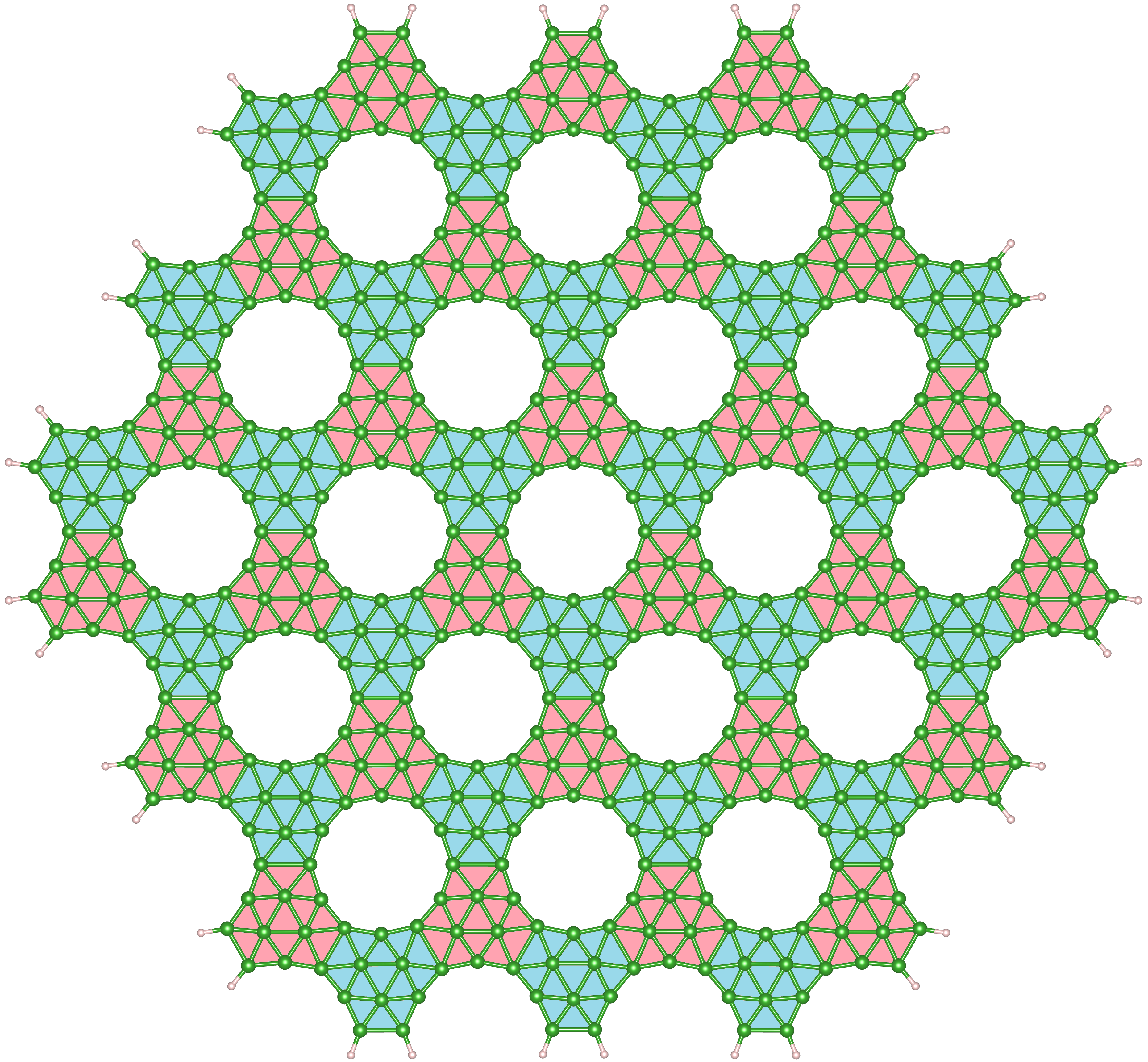


***Table S1.*** Total energies and Cartesian coordinates (in Angstroms) at the B3LYP/6-311++G(d,p) level of theory for the optimized structures shown in Figs. 1 and 2.

**B_12_ – 2D**

**Energy = -298.02684363 Hartree**

B -0.004936010 -2.423118430 -0.172061780

B 1.337437200 -1.528721130 -0.044776060

B -1.475213860 -1.921887960 -0.173723540

B -0.312964040 -0.918085020 0.392682860

B -1.992615410 -0.393761380 -0.046357500

B -0.638852490 0.729655660 0.390640890

B -2.096408210 1.216401970 -0.172806610

B -0.926687210 2.238772410 -0.173199360

B 2.401841390 -0.316921040 -0.175197610

B 0.951782480 0.188120430 0.392177840

B 2.101270430 1.207204900 -0.173045590

B 0.655345730 1.922339590 -0.044333520

**B_12_H – 2D**

**Energy = -298.62152474 Hartree**

B 0.791402080 0.538635940 0.364890580

B -0.147440610 2.112550890 0.105942630

B 1.444955170 1.983778040 -0.100551110

B -1.688577410 -1.010513530 -0.108315500

B 0.069891100 -0.972258770 0.325726940

B -0.885543050 0.458365380 0.473096780

B 2.287125320 0.687583190 -0.265441300

B 1.715178800 -0.810279360 -0.182132650

B -1.717601870 1.808290640 0.017623960

B -2.415575970 0.430824740 -0.088758990

B 0.933645480 -2.332238380 -0.192953740

B -0.697154010 -2.241126810 -0.379071060

H 1.542441780 -3.314353700 0.092890970

**B_12_H_2_ – 2D**

**Energy = -299.27542897 Hartree**

B 0.807499620 0.623247450 0.339665420

B -0.131752630 2.160626930 -0.002805360

B 1.475483310 2.064354610 -0.110423120

B -1.682864260 -0.948632370 -0.052406560

B 0.066195230 -0.942758570 0.211967360

B -0.857956490 0.514534400 0.235260090

B 2.353639830 0.780369690 -0.123865020

B 1.785230230 -0.719097620 -0.025222800

B -1.721259920 1.870645630 -0.103716630

B -2.421317950 0.480182120 -0.162190010

B 1.033688660 -2.196289660 -0.165571000

B -0.784379450 -2.337356500 0.014605630

H -1.077239840 -3.473698600 0.119036220

H 1.458307570 -3.265461120 -0.411443970

**B_12_H_3_ – 2D**

**Energy = -299.87751743 Hartree**

B 0.734249790 0.734016550 0.218285470

B -0.387539020 1.972561150 -0.149234460

B 1.314885940 2.295625410 -0.170957360

B -1.603638820 -1.199081760 0.217288030

B 0.143414300 -1.001530580 0.309091800

B -0.889145610 0.386680650 0.295506750

B 2.187793610 0.943514460 -0.476013800

B 1.741639790 -0.555798600 -0.247810770

B -1.938912280 1.564667580 -0.156236050

B -2.486792730 0.122691410 0.048680440

B 1.192737120 -2.127198040 -0.133370150

B -0.559298260 -2.467548860 0.190461870

H -0.751790710 -3.627710040 0.243892470

H 1.820215060 -3.111217880 -0.276797920

H 1.679797070 3.352277120 0.232516550

**B_12_H_4_ – 2D**

**Energy = -300.52630219 Hartree**

B 0.421454110 -0.895032160 -0.064877400

B -1.137188380 -1.680017560 0.011440500

B 0.285122690 -2.514762330 0.180738440

B -1.137040180 1.680443650 0.011159190

B 0.421765500 0.895052570 -0.063754310

B -1.055802970 -0.000054560 -0.258051090

B 1.870939740 -1.656850610 -0.057272800

B 1.818470300 -0.000548360 0.020888110

B -2.468229090 -0.775726850 0.008223220

B -2.467674000 0.775887450 0.008739520

B 1.871412800 1.655972120 -0.053768190

B 0.285113380 2.515559750 0.177712510

H 0.489699740 3.646937380 0.431034260

H 2.741542380 2.430913670 -0.231193010

H 2.739616090 -2.431691780 -0.242810200

H 0.486802080 -3.645860150 0.437077520

**B_12_H_5_ – 2D**

**Energy = -301.12666644 Hartree**

B -0.869975870 -0.553737580 -0.252848330

B -1.760713020 0.819900500 0.096196990

B -2.463927220 -0.614145970 0.130504750

B 1.573856570 1.025715880 -0.044732920

B 0.859470570 -0.488925980 -0.018598570

B -0.085277820 1.071466170 -0.082413700

B -1.714430610 -2.035079640 -0.230563920

B -0.010825120 -1.918395200 0.035526470

B -1.051616560 2.335345980 0.170559280

B 0.744743290 2.461507240 -0.045364370

B 1.615109990 -1.909250030 0.309287180

B 2.492538050 -0.339911750 -0.063045110

H 3.640124210 -0.556744820 -0.223170440

H 2.372317850 -2.706530900 0.732408850

H 1.103456280 3.582505760 -0.096203680

H -1.564635360 3.380254840 0.341944980

H -2.176477520 -2.969439110 -0.801486370

**B_12_H_6_ – 2D**

**Energy = -301.78553623 Hartree**

B 0.762727790 0.762727790 0.000000000

B -0.489298090 1.826085310 0.000000000

B 1.086029380 2.367246160 0.000000000

B -1.336787230 -1.336787230 0.000000000

B 0.279177750 -1.041905530 0.000000000

B -1.041905530 0.279177750 0.000000000

B 2.367246160 1.086029380 0.000000000

B 1.826085310 -0.489298090 0.000000000

B -2.124152110 1.507080620 0.000000000

B -2.593110000 -0.243094050 0.000000000

B 1.507080620 -2.124152110 0.000000000

B -0.243094050 -2.593110000 0.000000000

H -0.390136520 -3.762879250 0.000000000

H 2.219307760 -3.063680760 0.000000000

H -3.762879250 -0.390136520 0.000000000

H -3.063680760 2.219307760 0.000000000

H 3.453817280 1.543571490 0.000000000

H 1.543571490 3.453817280 0.000000000

**B_12_H_7_ – 2D**

**Energy = -302.36595425 Hartree**

B -1.038734180 -0.487572690 0.287021870

B -0.003797610 -1.832222340 0.476920220

B -1.721526070 -1.859736830 -0.092839830

B 1.659381380 0.957983420 -0.084743770

B 0.003125920 1.050510870 -0.014572720

B 1.036776270 -0.492898580 0.286815540

B -2.622609120 -0.378012020 -0.062264710

B -1.654138630 0.964845600 -0.084352580

B 1.713111040 -1.866906220 -0.092159360

B 2.621286420 -0.390514610 -0.062563670

B -0.878818000 2.420214740 -0.258742290

B 0.889704780 2.414696580 -0.258247170

H 1.337390620 3.502065430 -0.355634370

H -1.320831100 3.507782100 -0.356511470

H 3.791911200 -0.506768160 -0.166523500

H 2.174350400 -2.928691330 -0.296779980

H -3.794332850 -0.489562110 -0.165981950

H -2.187057840 -2.917766710 -0.298009720

H -0.006457800 -2.726891150 1.264149570

**B_12_H_8_ – 2D**

**Energy = -302.96705411 Hartree**

B 0.114408170 0.532483400 0.926199510

B -0.089148990 1.990843790 0.000000000

B -0.298444330 1.893481440 1.687158000

B 0.297287970 -0.968846750 -1.603494920

B -0.126917410 -0.893315880 0.000000000

B 0.114408170 0.532483400 -0.926199510

B 0.269630080 0.370561970 2.541143480

B 0.297287970 -0.968846750 1.603494920

B -0.298444330 1.893481440 -1.687158000

B 0.269630080 0.370561970 -2.541143480

B -0.221823220 -2.407981540 0.874540540

B -0.221823220 -2.407981540 -0.874540540

H -0.992913280 -3.158308110 -1.371920720

H -0.992913280 -3.158308110 1.371920720

H 0.540764940 0.583370600 -3.668141620

H -0.471639480 2.813171310 -2.397543290

H 0.540764940 0.583370600 3.668141620

H -0.471639480 2.813171310 2.397543290

H 0.612300660 -3.031389480 0.000000000

H 0.709198150 2.897795850 0.000000000

**B_12_ – 3D**

**Energy = -297.83072287 Hartree**

B 1.398909000 0.764546640 0.267895420

B 0.679930370 0.719512180 -1.234262310

B 0.976676170 -0.695714840 1.044115400

B -0.153495660 -0.748447620 -1.377632300

B 1.313879040 -0.719628320 -0.584561100

B -0.028762800 1.723933300 -0.024988860

B -1.398909000 -0.764546640 -0.267895420

B -0.679930370 -0.719512180 1.234262310

B -0.976676170 0.695714840 -1.044115400

B 0.153495660 0.748447620 1.377632300

B -1.313879040 0.719628320 0.584561100

B 0.028762800 -1.723933300 0.024988860

**B_12_H – 3D**

**Energy = -298.51228313 Hartree**

B -0.021429780 0.000004510 -1.660430620

B -1.471519050 0.000023490 -0.813241030

B 1.136171460 -0.820177300 -0.770737820

B -1.144955850 -0.843558160 0.636977920

B -0.430608390 -1.377315320 -0.777285440

B -0.430606680 1.377310990 -0.777262170

B -0.007378840 0.000006050 1.703373760

B 1.448924530 0.000009280 0.665042700

B -1.144968510 0.843559870 0.636951080

B 1.136158980 0.820183040 -0.770741650

B 0.465781570 1.353882400 0.664120320

B 0.465796430 -1.353933190 0.664148270

H -0.001365880 0.000004320 2.882847810

**B_12_H_2_ – 3D**

**Energy = -299.19416316 Hartree**

B 0.000000000 0.000000000 -1.782724510

B -1.407522580 0.000000000 -0.717882570

B 1.138709690 -0.827321010 -0.717882570

B -1.138881140 -0.827445580 0.719564550

B -0.434948400 -1.338633520 -0.717882570

B -0.434948400 1.338633520 -0.717882570

B 0.000000000 0.000000000 1.784748120

B 1.407734510 0.000000000 0.719564550

B -1.138881140 0.827445580 0.719564550

B 1.138709690 0.827321010 -0.717882570

B 0.435013890 1.338835080 0.719564550

B 0.435013890 -1.338835080 0.719564550

H 0.000000000 0.000000000 -2.961916390

H 0.000000000 0.000000000 2.964386100

**B_12_H_3_ – 3D**

**Energy = -299.83602943 Hartree**

B 0.032755770 -0.100808710 -1.752901730

B -1.411421610 -0.078751270 -0.686477430

B 1.162911430 -0.900870580 -0.647731510

B -1.136229010 -0.855255590 0.749127620

B -0.411269150 -1.412378820 -0.647722030

B -0.455174960 1.400952770 -0.801824830

B -0.002202280 0.006760010 1.806030640

B 1.421916890 -0.024051670 0.749120840

B -1.135534370 0.798394100 0.715390190

B 1.188094420 0.765885100 -0.686478250

B 0.449396730 1.313363300 0.715378800

B 0.442098210 -1.360665890 0.751056370

H 0.045005620 -0.138527470 -2.931930600

H -0.000415430 0.001282590 2.985935810

H -0.775962740 2.388171160 -1.365392930

**B_12_H_4_ – 3D**

**Energy = -300.49368420 Hartree**

B 0.261138440 -1.793571250 0.000000000

B -1.052734530 -0.931712940 -0.840859870

B 0.478877410 -0.671407390 1.355427180

B -1.634883670 0.374532940 0.000000000

B -1.052734530 -0.931712940 0.840859870

B 0.478877410 -0.671407390 -1.355427180

B -0.462973410 1.693580490 0.000000000

B 0.968753190 0.956188970 0.913465910

B -0.664154860 0.541639050 -1.332650780

B 1.416663920 -0.505629320 0.000000000

B 0.968753190 0.956188970 -0.913465910

B -0.664154860 0.541639050 1.332650780

H 0.436481510 -2.960698820 0.000000000

H -0.682169620 2.852806120 0.000000000

H 1.718136240 1.659135190 1.495234060

H 1.718136240 1.659135190 -1.495234060

**B_12_H_5_ – 3D**

**Energy = -301.13600616 Hartree**

B -0.352505210 0.630472910 1.330144910

B -0.399149310 -0.966878430 1.134785800

B -0.791732880 1.560394420 -0.135119570

B -0.855554020 -1.437433180 -0.524834250

B -1.632970870 -0.018562300 0.334806990

B 1.037729380 -0.182128910 1.226143520

B 0.492527440 -0.632024980 -1.351749550

B 0.540200620 0.957854720 -1.136321810

B 0.886069900 -1.566741200 0.102353370

B 0.951962920 1.450644940 0.510032160

B 1.571449580 0.019718060 -0.319123440

B -0.935273600 0.184591470 -1.274241290

H -1.371498760 2.585824590 -0.207957400

H -2.788127550 -0.009260730 0.585292340

H 1.576520000 2.394605420 0.845956300

H 1.489446240 -2.579804110 0.161061880

H -1.462550460 -2.385896020 -0.879351870

**B_12_H_6_ – 3D**

**Energy = -301.80047467 Hartree**

B -0.269115000 0.972768800 1.231807050

B 0.846908800 1.566574710 -0.064202750

B -0.140482240 -0.886524620 1.316009620

B 1.464925690 0.016418760 -0.741580130

B 1.336979730 0.117092200 0.971529030

B -0.884705200 1.296021610 -0.365118620

B 0.305737730 -0.870874040 -1.399237540

B -0.800942050 -1.576740110 -0.222615440

B 0.157134160 0.773643860 -1.478989380

B -1.574511410 -0.067279750 0.509234380

B -1.158968300 -0.081012820 -1.160373300

B 0.940183700 -1.307472850 0.016907560

H -0.423749460 1.586245080 2.228844040

H -0.311860680 -1.430007700 2.350426770

H 2.215761620 0.244553990 1.750577600

H 1.425233490 2.594670570 -0.041209070

H -2.654069260 -0.150554470 0.980730650

H -1.369861320 -2.606501400 -0.314471380

**B_12_H_7_ – 3D**

**Energy = -302.44171091 Hartree**

B -0.202530110 1.018695720 -1.245753580

B -0.579160840 1.649767880 0.443645050

B -0.465030830 -0.763090150 -1.257727590

B -0.992295300 0.190762140 1.406023610

B -1.560775670 0.317398890 -0.268232380

B 1.043750360 1.181528330 -0.087535250

B 0.317962350 -0.829304630 1.332534630

B 0.732118920 -1.590878250 -0.190939460

B 0.592724160 0.747599530 1.400286050

B 1.214672450 -0.155840390 -1.199019290

B 1.608714760 -0.201357700 0.529845370

B -1.010889080 -1.280401280 0.375093260

H -0.402816300 1.698170440 -2.190385640

H -0.879118110 -1.304828080 -2.223530550

H -2.684311110 0.501889410 -0.585807290

H -1.741555620 -2.192577250 0.547576400

H -0.946152310 2.754671680 0.637881210

H 1.994511570 -0.205665520 -2.085222070

H 1.147331740 -2.686295720 -0.332214490

**B_12_H_8_ – 3D**

**Energy = -303.11410520 Hartree**

B 0.912332360 1.524169310 0.138706210

B 1.273231310 0.000000000 0.930998430

B -0.912332360 1.524169310 0.138706210

B 0.000000000 -0.812876380 1.481203760

B 0.000000000 0.812876380 1.481203760

B 1.464494600 0.000000000 -0.753326260

B -0.912332360 -1.524169310 0.138706210

B -1.464494600 0.000000000 -0.753326260

B 0.912332360 -1.524169310 0.138706210

B 0.000000000 0.912448210 -1.276268590

B 0.000000000 -0.912448210 -1.276268590

B -1.273231310 0.000000000 0.930998430

H 0.000000000 -1.511984050 -2.295468470

H -2.469910620 0.000000000 -1.372241560

H 0.000000000 1.511984050 -2.295468470

H 2.469910620 0.000000000 -1.372241560

H 1.584432620 -2.493932010 0.174784840

H -1.584432620 -2.493932010 0.174784840

H 1.584432620 2.493932010 0.174784840

H -1.584432620 2.493932010 0.174784840

**B_12_F – 2D**

**Energy = -397.94236191 Hartree**

B 0.602187440 0.937062900 0.363435980

B -0.346143800 2.535922640 0.083455590

B 1.240018720 2.384756580 -0.127737200

B -1.848658570 -0.587505350 -0.120775870

B -0.137916750 -0.555098380 0.344327330

B -1.080469100 0.886529310 0.457393230

B 2.088438780 1.092439030 -0.279646720

B 1.502766410 -0.407394530 -0.167675580

B -1.917041050 2.230212830 0.023542880

B -2.607053990 0.851293810 -0.098210150

B 0.765227810 -1.972817950 -0.219421510

B -0.880375580 -1.825957830 -0.408541100

F 1.453965580 -3.092545220 0.083122760

**B_12_F_2_ – 2D**

**Energy = -497.92724149 Hartree**

B 0.828296700 1.249391050 0.035837810

B 0.000280400 2.893300660 0.002077650

B 1.595045010 2.711751030 0.029211200

B -1.852789430 -0.097817550 -0.034243580

B -0.000391920 -0.255513650 0.004746270

B -0.828031920 1.250127290 -0.016914410

B 2.437549340 1.404278560 0.037242620

B 1.853114260 -0.098595710 0.025706860

B -1.594316950 2.711533400 -0.031341820

B -2.436194880 1.404066370 -0.052497550

B 1.086278140 -1.518479270 -0.025693040

B -1.086341440 -1.517551290 0.024233250

F -1.295481460 -2.814753620 0.081866000

F 1.292789220 -2.815966660 -0.084763240

**B_12_F_3_ – 2D**

**Energy = -597.84372170 Hartree**

B 0.608153460 0.904214020 0.165380460

B -0.240822060 2.360069300 -0.170619690

B 1.488919170 2.448633540 -0.056296290

B -2.068647530 -0.528963060 -0.017011520

B -0.254618260 -0.677057510 0.159599010

B -1.044231580 0.865438910 0.082010730

B 2.166975630 0.969894640 -0.362616780

B 1.494182680 -0.462021870 -0.207125310

B -1.833010370 2.258474160 -0.299270010

B -2.654702520 0.942278140 -0.212021140

B 0.760992560 -1.916761250 -0.101246510

B -1.301876180 -1.942531420 0.110826720

F -1.551513290 -3.238431430 0.172954220

F 1.065550950 -3.195630010 -0.148122720

F 2.085141860 3.533420700 0.479699840

**B_12_F_4_ – 2D**

**Energy = -697.82630011 Hartree**

B 0.920355000 0.110537270 0.004103580

B 1.702648360 1.741950580 0.001976500

B 2.568423390 0.372558320 -0.001402480

B -1.702142500 1.742347850 0.001779700

B -0.920418140 0.110764270 0.004049680

B 0.000242360 1.588471110 0.006879840

B 1.618016860 -1.362186480 0.001913810

B -0.000159820 -1.287348670 0.002436970

B 0.772698230 3.039781990 0.000996800

B -0.771863780 3.039904580 0.000904460

B -1.618311670 -1.361830730 0.002245580

B -2.568252350 0.373206980 -0.001641740

F -3.849664390 0.030879140 -0.008200250

F -2.572200370 -2.275619040 0.001874940

F 2.571537410 -2.276359390 0.001307420

F 3.849734990 0.029728410 -0.007463670

**B_12_F_5_ – 2D**

**Energy = -797.74858166 Hartree**

B -1.141591260 -0.575870090 -0.088878750

B -2.040332040 0.780200000 0.072452660

B -2.783708680 -0.647243650 0.078801180

B 1.292934650 0.972721830 0.006307450

B 0.648977300 -0.575287720 0.029999680

B -0.362013290 1.074428740 -0.029593370

B -1.987654220 -2.112692160 -0.179090200

B -0.288710260 -1.953028110 -0.000529040

B -1.424999840 2.301545360 0.097313520

B 0.534947680 2.411180940 -0.032418580

B 1.329452550 -2.060317630 0.142552090

B 2.258615620 -0.340455480 0.065256770

F 3.557100750 -0.595313850 0.085178520

F 2.250878750 -2.994551400 0.310713130

F 0.842337250 3.697552230 -0.076349810

F -1.874960270 3.540527150 0.191168440

F -2.575289170 -3.242775210 -0.601546550

**B_12_F_6_ – 2D**

**Energy = -897.73726236 Hartree**

B 2.617695790 0.001786450 0.000000000

B 1.470503420 1.177377970 0.000000000

B 1.924623870 -1.774303580 0.000000000

B 1.042578180 -0.406847080 0.000000000

B 0.284387520 -1.862182310 0.000000000

B -0.873628990 -0.699475650 0.000000000

B -1.307300780 -2.267884280 0.000000000

B -2.498903910 -0.779621380 0.000000000

B 0.574280030 2.553924960 0.000000000

B -0.168949180 1.106322730 0.000000000

B -1.310395000 2.266097830 0.000000000

B -1.754890940 0.684804340 0.000000000

F -1.937372620 3.434039430 0.000000000

F 0.824173150 3.855727060 0.000000000

F 3.942651690 -0.039205810 0.000000000

F 2.927071010 -2.641618420 0.000000000

F -2.005279080 -3.394833620 0.000000000

F -3.751244170 -1.214108640 0.000000000

**B_12_F_7_ – 2D**

**Energy = -997.61199841 Hartree**

B -1.122192310 -0.442639690 0.390807830

B -0.003909570 -1.649966150 0.555667060

B -2.020667920 -1.631540720 -0.178382500

B 1.625178000 1.034923170 -0.029033840

B 0.003011300 1.218819300 -0.029244400

B 1.119917140 -0.447740040 0.390934590

B -2.780305200 -0.114907280 0.136928380

B -1.620388890 1.042311740 -0.029230990

B 2.013177700 -1.640816220 -0.179374720

B 2.779859980 -0.128138740 0.137247540

B -0.910532580 2.518066660 -0.298319320

B 0.921872050 2.513734920 -0.297779200

F 1.426096310 3.717129890 -0.554058750

F -1.408468420 3.723671330 -0.554914210

F 4.100293190 -0.053510720 0.374322970

F 2.340132470 -2.855264560 -0.519655500

F -4.100142700 -0.034994260 0.372730940

F -2.353774830 -2.844747630 -0.518006390

F -0.006492300 -2.894282030 1.050075630

**B_12_F_8_ – 2D**

**Energy = -1097.56845952 Hartree**

B 1.007882180 0.582126200 0.374060110

B -0.003191310 2.244829380 0.736978060

B 1.436874170 2.073250550 -0.127918150

B -1.572546720 -1.013673370 0.134577800

B 0.005948040 -0.615724450 -0.099848780

B -1.016843030 0.573478380 0.367289570

B 2.393921130 0.241418700 -0.493042290

B 1.581482080 -1.003730950 0.135045520

B -1.443818770 2.066479250 -0.137461110

B -2.389225210 0.237545490 -0.490344960

B 1.322609180 -2.571527840 0.323996490

B -1.309435160 -2.586817640 0.327792670

F -1.716749520 -3.724681630 -0.147941690

F 1.728492850 -3.713985300 -0.152466200

F -3.527388400 0.572447260 -1.074099860

F -2.377856940 2.950328050 -0.443683920

F 3.516454670 0.592871820 -1.072805750

F 2.358798970 2.968364300 -0.443796790

F 0.014965550 -2.940427130 1.124098860

F -0.010030140 3.057076970 1.848423160

**B_12_F – 3D**

**Energy = -397.84061013 Hartree**

B 0.017292620 0.000066880 -2.021041570

B -1.397048270 0.000021900 -1.121024160

B 1.175839160 -0.866410500 -1.153592380

B -1.169965180 -0.835340970 0.309729340

B -0.446288560 -1.342910000 -1.122133360

B -0.446541350 1.343288760 -1.122378940

B -0.000441810 0.000089530 1.342103090

B 1.415135060 0.000081260 0.277510040

B -1.169899200 0.835313660 0.309674280

B 1.176098870 0.866495190 -1.154115840

B 0.422072980 1.362784890 0.297587430

B 0.422002460 -1.362737280 0.298017450

F 0.000756570 -0.000343900 2.699526070

**B_12_F_2_ – 3D**

**Energy = -497.84910692 Hartree**

B 0.000000000 0.000000000 1.770096940

B -1.407163470 0.000000000 0.717227310

B 1.138419160 0.827109930 0.717227310

B -1.138691720 0.827307960 -0.715742930

B -0.434837420 1.338291980 0.717227310

B -0.434837420 -1.338291980 0.717227310

B 0.000000000 0.000000000 -1.768771590

B 1.407500370 0.000000000 -0.715742930

B -1.138691720 -0.827307960 -0.715742930

B 1.138419160 -0.827109930 0.717227310

B 0.434941530 -1.338612400 -0.715742930

B 0.434941530 1.338612400 -0.715742930

F 0.000000000 0.000000000 3.126116600

F 0.000000000 0.000000000 -3.124607470

**B_12_F_3_ – 3D**

**Energy = -597.80795216 Hartree**

B 0.093324980 -0.287205770 -1.624995790

B -1.340594210 -0.287822730 -0.544697590

B 1.231034430 -1.112951320 -0.555640740

B -1.045250010 -1.092561630 0.853284980

B -0.341774840 -1.623980260 -0.555659330

B -0.392866650 1.209087170 -0.630551740

B 0.085563740 -0.263258940 1.917062600

B 1.487831510 -0.269508680 0.853267040

B -1.056636580 0.556569900 0.866380540

B 1.253780010 0.555126820 -0.544669340

B 0.527677910 1.071329520 0.866381010

B 0.529458170 -1.629430230 0.839284270

F 0.100530340 -0.309379510 3.272056990

F 0.090203670 -0.277609250 -2.975777150

F -0.763503180 2.349841790 -1.262251270

**B_12_F_4_ – 3D**

**Energy = -697.77978227 Hartree**

B 0.201989520 -2.071623640 0.000000000

B -1.184637340 -1.332530500 -0.844863980

B 0.321644220 -0.946718620 1.356383590

B -1.888447080 -0.095773240 0.000000000

B -1.184637340 -1.332530500 0.844863980

B 0.321644220 -0.946718620 -1.356383590

B -0.846617180 1.316571790 0.000000000

B 0.675782270 0.705740570 0.928686760

B -0.933246240 0.153092350 -1.344798110

B 1.237473600 -0.709091880 0.000000000

B 0.675782270 0.705740570 -0.928686760

B -0.933246240 0.153092350 1.344798110

F 0.491773990 -3.397345970 0.000000000

F -1.180319620 2.622366930 0.000000000

F 1.448263630 1.606009020 1.580482760

F 1.448263630 1.606009020 -1.580482760

**B_12_F_5_ – 3D**

**Energy = -797.74700606 Hartree**

B 0.016645800 -0.784643010 1.227880110

B 0.063760100 0.804918700 1.208154730

B 0.605066980 -1.534284060 -0.297624670

B 0.699528140 1.484954270 -0.331723050

B 1.403111640 -0.041554630 0.448376190

B -1.352018820 0.053853090 1.031173590

B -0.558961780 0.797573610 -1.377863480

B -0.605401340 -0.793030160 -1.362578830

B -1.121530120 1.546242220 0.122727750

B -1.214082430 -1.472047760 0.155431350

B -1.767859640 0.049496320 -0.584342230

B 0.860279350 -0.042742940 -1.246506710

F 1.414279710 2.614739100 -0.511970010

F -1.786290630 2.715378890 0.264577890

F 2.690771250 -0.076498400 0.867894220

F 1.246528940 -2.710343060 -0.457406650

F -1.953043170 -2.592166430 0.322388370

**B_12_F_6_ – 3D**

**Energy = -897.71427081 Hartree**

B -0.184974350 0.977687110 0.699472960

B 0.932373250 1.559303670 -0.613931370

B -0.052614360 -0.923323320 0.790112490

B 1.565455270 -0.021550050 -1.275326530

B 1.445544080 0.095051730 0.435997220

B -0.817786840 1.284356050 -0.900924920

B 0.391675350 -0.897184010 -1.909616730

B -0.713384540 -1.609648800 -0.760238550

B 0.230818890 0.754133070 -1.995712460

B -1.510691270 -0.080289710 -0.028695620

B -1.091710750 -0.087413610 -1.696394050

B 1.042215520 -1.335966030 -0.508418810

F -0.363291550 1.642292210 1.860121140

F -0.250694180 -1.501321070 1.993153840

F 2.424065580 0.250399880 1.354785260

F 1.604441810 2.728423220 -0.591409440

F -2.727747860 -0.180853680 0.549691090

F -1.372369820 -2.781816680 -0.864461300

**B_12_F_7_ – 3D**

**Energy = -997.67033278 Hartree**

B 0.062893030 1.152617750 -0.807283520

B -0.321303420 1.768555850 0.887814890

B -0.212425760 -0.678504760 -0.823348600

B -0.727041690 0.274901330 1.830791680

B -1.332068840 0.427423730 0.189209780

B 1.351592500 1.333532260 0.345563720

B 0.557248450 -0.738292260 1.809491870

B 0.988296680 -1.505394630 0.265246880

B 0.826904230 0.846475180 1.812586540

B 1.479029140 -0.043329330 -0.761898270

B 1.834077360 -0.088510830 0.946083730

B -0.776813390 -1.200923900 0.825719970

F -0.217494280 1.887751010 -1.903386500

F -0.708813160 -1.268238810 -1.935659550

F -2.602807080 0.630591240 -0.220890490

F -1.623030120 -2.238616480 1.003721360

F -0.759813780 3.020063920 1.136999840

F 2.362385650 -0.142776910 -1.779318810

F 1.478769510 -2.747453130 0.089128800

**B_12_F_8_ – 3D**

**Energy = -1097.67079658 Hartree**

B 0.931714090 -1.557452990 -0.534099780

B 1.309374440 -0.035628450 -1.373151850

B -0.913126620 -1.562204700 -0.551825380

B 0.016555910 0.759698900 -1.926368340

B 0.020381710 -0.866893720 -1.880513590

B 1.486892230 0.012777820 0.329949350

B -0.920322330 1.524321040 -0.638795380

B -1.492915810 0.004979530 0.301263680

B 0.924490160 1.529187860 -0.621063690

B -0.005871380 -0.914456910 0.861927160

B -0.010272710 0.961457940 0.809035210

B -1.282436880 -0.042418390 -1.398043130

F -0.023386150 1.628958250 1.985388310

F -2.617839020 0.023358750 1.042146860

F -0.015960080 -1.514760300 2.073988320

F 2.597304770 0.036966170 1.092295050

F 1.682062380 2.641477360 -0.688585480

F -1.682339050 2.632578610 -0.720796200

F 1.694503100 -2.668229440 -0.539047240

F -1.669908590 -2.676899160 -0.571333010
